# Sentinel lymph node biopsy in periocular merkel cell carcinoma: a case report

**DOI:** 10.1186/s13104-017-2746-y

**Published:** 2017-09-20

**Authors:** Dan C. Filitis, Gyorgy Paragh, Faramarz H. Samie, Nathalie C. Zeitouni

**Affiliations:** 1 Department of Dermatology, Columbia University Medical Center, 161 Fort Washington Avenue, 12th Floor, New York, NY 10032 USA; 2 Department of Dermatology, Department of Cell Stress Biology, Roswell Park Cancer Institute, Buffalo, USA; 3 Columbia University Medical Center, New York, USA; 4 University of Arizona COM Phoenix, University of Arizona Cancer Center at Dignity Health, 625 N 6th Street, Phoenix, AZ 85004 USA

**Keywords:** Merkel cell carcinoma, Periocular, Sentinel lymph node biopsy, Case report

## Abstract

**Background:**

The National Comprehensive Cancer Network guidelines for Merkel cell carcinoma recommend performance of the sentinel lymph node biopsy in all patients with clinically negative nodal disease for staging and treatment. Nevertheless, sentinel lymph node biopsy in the periocular region is debated as tumors are typically smaller and lymphatic variability can make performance procedurally problematic.

**Case presentation:**

We present a case of a Caucasian patient in their seventies who presented with a 1.0 cm periocular Merkel cell carcinoma, who underwent Mohs surgery with a Tenzel flap repair, that was found to have a positive sentinel lymph node biopsy, but who, despite parotidectomy, selective neck dissection, and radiation, succumbed to the disease.

**Conclusions:**

Evidence in both the site-specific and non-specific literature demonstrates: (1) Worsening prognosis with extent of lymph node burden, (2) improvements in our abilities to perform lymphoscintigraphy, (3) locoregional and distant metastatic disease in patients with tumor sizes ≤1 cm, and (4) significant rates of sentinel lymph node positivity in patients with tumor sizes ≤1 cm. Our case supports that sentinel lymph node biopsy should be considered in all clinically nodal negative periocular Merkel cell carcinoma, regardless of size, and despite limited site-specific studies on the subject.

## Background

The sentinel lymph node biopsy (SLNB) plays an important role in the current staging and treatment of Merkel cell carcinoma (MCC) [1]. The National Comprehensive Cancer Network (NCCN) guidelines reflect this, recommending performance of the SLNB in all MCC patients that are clinically negative for nodal disease [2]. Still, whether all MCC patients are candidates for a SLNB, regardless of site of presentation or tumor characteristics, remains investigational and a matter of debate [3].

Periocular MCC represents anywhere from 5 to 20% of head and neck MCC [4, 5]. This is a unique site with a high degree of lymphatic drainage variability where tumors are often diagnosed earlier at smaller tumor sizes [6, 7]. Herein, we present a case of periocular MCC that highlights the aforementioned factors, and through review of the existing literature, aim to provide context for the decision to pursue SLNB at this distinctive site.

## Case presentation

A Caucasian patient in their seventies with no personal or familial history of cutaneous malignancy presented with a biopsy-proven MCC, with angiolymphatic invasion of the right upper eyelid. Immunohistochemistry was positive for CK20 and negative for TTF-1. The patient underwent Mohs micrographic surgery and SLNB. At this time the small violaceous plaque measured 1.0 × 0.8 cm and clinically and histologically residual MCC was noted in the tumor-debulking specimen. After two stages with negative margins, the final surgical defect measured 2.2 × 0.8 cm. The primary site was reconstructed with a Tenzel flap immediately following Mohs surgery. The SLN was positive for metastatic MCC and the patient thereafter underwent right superficial parotidectomy with facial nerve dissection and right selective neck dissection of levels 1–4. The parotid gland and all lymph nodes removed were negative for lymph node metastasis. Eight months after initial diagnosis the patient presented with multiple subdermal sub-centimeter masses palpable in the soft tissues of the right neck. Fine needle aspiration of a representative lesion was consistent with metastatic MCC. CT of the neck and chest with contrast identified diffuse metastatic disease affecting the lungs, liver, and the adrenals. The patient started palliative radiation for the right neck but succumbed to widespread metastatic disease 1 month later.

## Conclusions

Periocular MCC is rare, and as such, literature and data are scarce. In fact, much of what we believe about the tumor’s behavior and the role for SLNB at this site is extrapolated from MCC at other anatomic locations and data from other eyelid and conjunctival tumors.

Due to large variation in lymphatic drainage, higher rates of false negatives have been associated with SLNB in the head and neck region and the periocular region is no exception [7, 8]. In the periocular region, previous false-negative values in studies looking at sebaceous carcinoma and melanoma ranged from 11 to 20% [9]. More recently however, rates of successful identification of SLN, on either pre- or intraoperative lymphoscintigraphy, were found to be as high as 97 and 100%, respectively [10–13].

Site-nonspecific MCC studies report 11–57% SLN positivity [1, 4, 14–25]. SLN positivity showed positive correlation with tumor size, tumor depth, mitotic rate, histological growth pattern, and presence lymphovascular invasion [4, 14–16]. The literature on tumor factors associated with SLN positivity in periocular tumors is limited but trends suggest correlation of nodal metastasis with increased tumor size. In a recent study, 3 of 4 patients presenting with nodal metastasis were found to have >T2b tumors at presentation, suggesting more nodal involvement with larger tumor size as has been demonstrated in the site-nonspecific literature [26].

Still, in comparison to MCC at other sites, for eyelid specific disease, performance of SNLB is seldom reported to date (Table 1). Amato et al. reported on a 61-year-old male with a right upper eyelid lesion that had a positive SLNB. Tumor characteristics were not provided [13]. Maalouf et al. [11] performed SLNB in 4 patients with periocular MCC, all with tumors ≤2 cm, and found 1 of 4 to have a positive SLNB. One of the 3 patients with negative SLNB was defined as having had a falsely negative SLNB as they developed nodal disease 6 months after surgery. The median follow-up in that study was 18.7 months. Sneigowski et al. [26] performed SLN mapping in just 3 of 18 patients in their study—the demographics and tumor characteristics of which were not provided. Two of these patients had positive findings. Peters et al. reported performance of a SLNB in a 52-year-old woman with 1 cm-sized lesion on the left upper eyelid. This was found to be negative [27]. Herbert et al. [28] reported performance of a SLNB in just 1 patient of their 21. Tumor size at presentation was <1 cm, and the results of SNLB were negative. Finally, Esmaeli et al. [29] reported a positive SLNB in a 61-year-old man with a 1.2 cm lesion on the left upper eyelid.

**Table 1:** Outcomes of sentinel lymph node biopsy in periocular merkel cell carcinoma

Study	# of patients	Age/gender	Tumor size	SLNB	Locoregional metastasis
Amato et al. [13]	1	61/male	NA	+	NA
Maalouf et al. [11]	4	NA	≤ 2 cm	+	NA
				−	Yes
				−	NA
				−	NA
Sniegowski et al. [26]	3	NA	NA	+	NA
				+	NA
				−	NA
Peters et al. [27]	3	52/female	1 cm	−	NA
		96/male	1 cm	NA	Yes
		74/male	0.6 cm	NA	Yes
Herbert et al. [28]	2	NA	< 1 cm	−	NA
		69/female	0.8 cm	NA	Yes
Esmaeli et al. [29]	1	61/male	1.2 cm	+	NA
Kivelä et al. [5]	1	79/female	0.8 cm	NA	Yes
Filitis et al. *	1	seventies/NA	1 cm	+	Yes

*NA* not available/reported/performed

* current study

Some of these same studies report regional nodal and metastatic spread tends to be lower than at other sites at around 19–22% [26–28]. Some authors speculate that decreased tumor size at presentation may explain these lower reported rates of nodal metastasis [30]. Site-associated variation in lesion size has been previously demonstrated and some authors have attributed it to earlier detection. Yin et al. [6] speculate that earlier detection in eyelid disease specifically may be due to better visualization and perhaps related to visual field impedance, as that would be difficult to disregard. In a study by Allen et al. [22] the authors showed that the head and neck region, compared to other sites, demonstrates significantly smaller tumor diameters, as well as decreased clinically apparent nodal disease. Taken together, site associated tumor size and trends between size and SLN positivity may explain the decreased nodal metastatic potential seen in some periocular disease.

Even smaller MCC carry a significant risk of loco-regional disease spread. For example, Schwartz et al. demonstrated SLNB positivity rates of 23% in tumor sizes ≤1 cm, while Fields et al. showed a similar 26% SLN positivity at ≤1 cm tumor size, and Iyer et al. demonstrated 14% nodal involvement for 0.5 cm tumors [14–16]. In other cutaneous malignancies a threshold of 10% nodal positivity is widely used to justify performance of SLNB [4]. To date, the overwhelming majority of studies looking at SLN positivity in MCC demonstrate SNL positivity rates above this threshold, even in small tumors.

Cases reported in the periocular-specific MCC literature demonstrate regional nodal and/or distant metastatic disease at tumor sizes ≤1 cm [5, 27, 28] (Table 1). Kivelä et al. reported a case of a 79-year-old female with a 0.8 cm tumor that developed regional nodal metastasis approximately 6 months after biopsy and radiation [5]. Peters et al. reported 2 cases with tumor sizes ≤1 cm that went on to have nodal metastasis; one, a 96-year-old male, who developed ipsilateral submandibular node involvement 2.5 years after Mohs with 3 mm margins for a 1 cm left canthus lesion, and two, a 74-year-old male who developed ipsilateral parotid gland recurrence after 12 mm wide excision of a 4 × 6 mm right upper eyelid lesion [27]. Herbert et al. reported a 69-year-old female who presented with 8 mm eyelid lesion that developed both regional metastasis to the parotid nodes and distant metastasis to the chest 6 months after wide local excision. [28].

The case we present further exhibits the unpredictability of clinical outcomes with small periocular MCC—presenting at 1.0 cm on the right upper eyelid. Performance of lymphoscintigraphy and SLNB, as seen in our case, demonstrates how SNL positivity can be a helpful towards understanding the severity of one’s disease and justifying additional intervention. To this end, in the recent past, Iyer et al. [14] demonstrated that more extensive lymph node involvement is correlated with decreased survival, and just this past year, taking advantage of the tumor’s programmed-death-ligand-1 (PD-L1) expression, a report showed a 56% objective response rate when patients with advanced MCC were treated with pembrolizumab [31]. In March 2017, the United States Food and Drug Administration (FDA) granted accelerated approval of avelumab, an anti-PD-L1 antibody and the first FDA-approved medication to treat metastatic MCC [32]. With the advent of immunotherapy early accurate risk assessment may translate into better survival even if this has previously only been a distant mirage.

Evidence clearly outlining a role for SLNB in periocular MCC is largely lacking, nevertheless, implications from the general MCC literature, periocular-specific studies, and case reports such as ours, are relevant and worth consideration. Collectively, this data, and now the development of new, risk-stratified, treatment options help explain why multiple authors, ourselves included, continue to advocate that SLNB be strongly considered, [33] if not recommended, [30] for all periocular MCC and regardless of size.

## Abbreviations

MCC: Merkel cell carcinoma
NCCN: National Comprehensive Cancer Network
PD-L1: programmed death ligand 1
SLN: sentinel lymph node
SLNB: sentinel lymph node biopsy
FDA: Food and Drug Administration
